# SVDNVLDA: predicting lncRNA-disease associations by Singular Value Decomposition and *node2vec*

**DOI:** 10.1186/s12859-021-04457-1

**Published:** 2021-11-02

**Authors:** Jianwei Li, Jianing Li, Mengfan Kong, Duanyang Wang, Kun Fu, Jiangcheng Shi

**Affiliations:** 1 Institute of Computational Medicine, School of Artificial Intelligence, Hebei University of Technology, Tianjin, 300401 China; 2 Hebei Province Key Laboratory of Big Data Calculation, Hebei University of Technology, Tianjin, 300401 China; 3 School of Life Sciences, Tiangong University, Tianjin, 300387 China

**Keywords:** LncRNA-disease association prediction, Singular Value Decomposition, *node2vec*, Network representation learning, XGBoost classifier

## Abstract

**Background:**

Numerous studies on discovering the roles of long non-coding RNAs (lncRNAs) in the occurrence, development and prognosis progresses of various human diseases have drawn substantial attentions. Since only a tiny portion of lncRNA-disease associations have been properly annotated, an increasing number of computational methods have been proposed for predicting potential lncRNA-disease associations. However, traditional predicting models lack the ability to precisely extract features of biomolecules, it is urgent to find a model which can identify potential lncRNA-disease associations with both efficiency and accuracy.

**Results:**

In this study, we proposed a novel model, SVDNVLDA, which gained the linear and non-linear features of lncRNAs and diseases with Singular Value Decomposition (SVD) and node2vec methods respectively. The integrated features were constructed from connecting the linear and non-linear features of each entity, which could effectively enhance the semantics contained in ultimate representations. And an XGBoost classifier was employed for identifying potential lncRNA-disease associations eventually.

**Conclusions:**

We propose a novel model to predict lncRNA-disease associations. This model is expected to identify potential relationships between lncRNAs and diseases and further explore the disease mechanisms at the lncRNA molecular level.

**Supplementary Information:**

The online version contains supplementary material available at 10.1186/s12859-021-04457-1.

## Background

Since the central dogma of molecular biology was proposed, RNA has been treated as an intermediary between protein-coding gene and protein. However, protein-coding genes account for only ~ 1.5% of the human genome, and more than 98% of the human genome cannot encode proteins [1–3]. Most non-coding genes would be transcribed into non-coding RNAs (ncRNAs). As their names imply, ncRNAs cannot be directly translated into proteins, so they were often considered as the "noise" of genome transcription without any biological functions for decades. According to the lengths of nucleotide sequences, ncRNAs can be further divided into small ncRNAs (< 200 nucleotides) and long ncRNAs (> 200 nucleotides) [4, 5]. Following the discovery of lncRNA H19 and XIST in the early 1990s [6, 7], associated with the rapid developments of scientific methodologies and experimental techniques, researchers have identified thousands of lncRNAs in eukaryotes ranging from nematodes to humans [8, 9]. Abundant evidences have demonstrated that lncRNAs play important roles in many fundamental and critical biological processes, such as transcriptional and post-transcriptional regulation, epigenetic regulation and chromosome dynamics [10–14]. Previous studies showed that the mutation or dysregulation of lncRNAs are closely related with a variety of human diseases. For instance, MALAT1, also known as NEAT2, was found upregulated in non-small cell lung cancer tissues and could be served as an early prognostic biomarker [15]; lncRNA HOTAIR had been explored as a potential biomarker on the detection of hepatocellular carcinoma relapse [16].

The complex and precise regulatory functions of lncRNAs have largely explained the complexity of genome and opened a new chapter for scientists to deeply understand the diversity of living organisms from the perspective on gene expression regulatory network. However, the exact mechanisms behind these various regulative relationships remain to be further explored; the general characteristics of lncRNAs, such as the relationships between their spatial structures and functions, the realization of transcriptional regulation, and the molecular level mechanisms in various biological processes or diseases, are still unknown. The identification of lncRNA-disease associations can not only help us better understand the underlying mechanisms of lncRNAs in various human diseases, but also accelerate the discovery of potential biomarkers which may benefit the diagnosis, treatment, prognosis of many complex diseases. The exploration on the association between lncRNA and disease has attracted more and more researchers’ attention nowadays, which has become a prevalent topic in the current research field of lncRNA. Due to the number of newly discovered lncRNAs is growing rapidly every year, identifying lncRNA-disease association purely based on clinical information and biological experiments has encountered bottlenecks for their enormous consume of time and cost, and their disability to predict the associations of unrecorded diseases or lncRNAs, which undoubtedly limits the development of the lncRNA related studies. However, computational methods based on biological data can rapidly and efficiently quantify the correlation probability of interested lncRNA-disease pairs automatically, which can significantly reduce the time and cost of biological experiments. Therefore, it is a significant and urgent task to develop efficient and robust computational methods that are capable for predicting potential lncRNA-disease associations and providing candidates for future experimental verification.

Many researchers have proposed numerous algorithms and models for predicting potential lncRNA-disease association relationships over the years. All these methods could be broadly divided into three groups: biological network-based methods, machine learning-based methods and others. Based on the hypothesis that lncRNAs with similar functions may be more likely to be associated with diseases with similar phenotypes [17], a significant number of different biological network-based methods have been proposed by integrating multi-source biological information networks to detect potential disease-related lncRNAs. Sun et al. [18] proposed a global network-based computing method, RWRLNCD. By integrating a lncRNA-disease association network and a disease similarity network into a lncRNA functional similarity network, RWRLNCD adopted the Random Walk with Restart (RWR) algorithm on the constructed lncRNA functionally similar network to conduct predictions. Yao et al. [19] proposed a predictive model named LNCPricNet, which was based on a multi-layer composite network fusing different data of phenotypic-phenotypic interactions, lncRNA-lncRNA interactions and gene–gene interactions with disease-ncRNA relationships. The RWR algorithm was applied to predict potential lncRNA-disease associations. LNCPricNet could still achieve a decent performance when the known lncRNA-disease association data was insufficient, which may largely thank to the fact that the multi-layer composite network interacted with abundant information offseted the insufficient with one particular type of data. Ding et al. [20] came up with a model named TPGLDA in which built a lncRNA-disease-gene tripartite graph and applied a resource allocation algorithm to obtain the promising lncRNA-disease associations. Zhao et al. [21] built a multi-heterogeneous network which integrated the lncRNA functional similarity network, genetic similarity network, disease semantic similarity network and association networks among these three kinds of biological entries, subsequently realized the prediction of underlying lncRNA-disease associations through the RWR algorithm on their heterogeneous network. Xie et al. [22] adopted unbalance bi-random walk in their heterogeneous network to reconstruct the lncRNA-disease association matrix, which reflected the latent lncRNA-disease associations. After that, they proposed a NCPHLDA model [23], which constructed two cosine similarity networks for all lncRNAs and diseases separately, and combined the network consistency projection score for each similarity network as the associated probability of corresponding lncRNA-disease pairs. Most of these biological network-based methods adopted random walk-based algorithms on the established heterogeneous networks, which essentially takes the underlying topology information of nodes in the heterogeneous networks as the basis for the potential association prediction. The predicted effects of network-based methods heavily depend on whether the built network could accurately and comprehensively reflect the interactions among real biomolecules. Meanwhile, the rigid neighborhood relationship utilized by the random walk algorithm or its derivations limits the information richness of molecular features.

In recent years, machine learning and deep learning techniques have been widely adopted in lncRNA-disease assocaition predictions. Most of machine learning methods for disease-related lncRNA candidate selection typically train classifiers with the acquired features of experimentally confirmed lncRNA-disease associations and interested candidates, then rank the candidating associations according to the classification results. Chen et al. [17] came up with a calculating model, LRLSLDA (Laplacian Regularized Least Squares for lncRNA-Disease Association), based on the “guilt by association” assumption that similar diseases tend to be associated with lncRNAs which possess similar functions. They developed a semi-supervised learning framework to predict potential disease-lncRNA associations. However, there are too many parameters involved in their model, and how to adjust parameters was not well addressed. In addition, the same lncRNA-disease pairs may get different scores from the lncRNA space and the disease space respectively, how to properly combining these scores is a tricky problem. Liu et al. [24] designed a computational model by integrating known human disease genes, human lncRNAs and gene expression profiles without relying on any known human lncRNA-disease relationships. However, this model could not predict disease-associated lncRNAs which have no associated gene records. Guo et al. [25] integrated the Gaussian interaction profile kernel similarity of lncRNAs and diseases with disease semantic similarity, and utilized an autoencoder getting lower-dimensional features of lncRNA-disease pairs. Finally, a rotating forest classifier was adopted to gain the prediction results. Beyond that, several deep learning-based models have been developed in lncRNA-disease prediction field. Zeng et al. [26] initially combined matrix factorization method with a two-hidden-layer neural network architecture to capture the linear and non-linear features of lncRNAs and diseases respectively. Subsequently, they proposed a deep learning framework named DMFLDA [27], which adopted deep matrix factorization to learn the represents of lncRNAs and diseases. Besides, they also proposed a SDLDA model [28] mixed matrix factorization method with neural network framework to extract different features of lncRNAs and diseases.

In addition to biological networks and machine learning methods, plenty of statistical methods are also adopted to predict latent lncRNA-disease associations. Chen et al. [29] proposed a HGLDA model based on hypergeometric distribution, where the functional similarity of lncRNA was calculated by integrating disease semantic similarity, miRNA-disease association, and miRNA-lncRNA interaction. By testing whether the number of the common miRNAs shared by the disease and the lncRNA which were in the same lncRNA-disease pair exteeded beyond some threshold, HGLDA performed hypergeometric distribution tests for each lncRNA-disease pair. Lu et al. [30] proposed a matrix factorization-based model, SIMCLDA. According to known lncRNA-disease, gene-disease, gene–gene interactions and the functional similarities of diseases, the Gaussian interaction kernel of lncRNAs was calculated, the matrix decomposition method was introduced to predict the potential lncRNA-disease associations. However, it did not tackle the problem of data sparsity and further studies are needed to improve its performance. Apart from statistical methods, there are still a lot of novel algorithms could be applied for potential association predictions. For example, Fan et al. [31] introduced graph convolutional matrix completion to implement potential lncRNA-disease associations. Fusing verified lncRNA-disease associations and similarity data, they constructed an encoder-decoder model to learn nodes embeddings and score associations respectively.

In this paper, we propose an integrated feature extraction model, Singular Value Decomposition SVD and Node2Vec based LncRNA-Disease Association prediction model (SVDNVLDA), to predict potential lncRNA-disease associations. The rest of this paper is arranged as follows:

The results and discussions section exhibits the influences of hyperparameters in SVDNVLDA, the results of model comparison, robustness test and case studies, as well as an in-depth analysis of the limitions of SVDNVLDA and futher improvement directions.

The conclusion section overviews the workflow of SVDNVLDA, and its first-class prediction capabality for practical applications.

The methods section introduces the acquisition and preprocessing of experimental data, the prediction process of SVDNVLDA, and the theoretical datails of SVD and node2vec methods involved in our model.

## Results and discussions

### Evaluation metrics

Except for special instructions, all the numerical experimental results involved in this paper were generated under tenfold cross-validations. The evaluation metrics used in classifier selection and parameter adjustment processes contained Accuracy (*Acc*), Sensitivity (*Sen*), Specificity (*Spec*), Precision (*Prec*), and Matthews correlation coefficient (*MCC*) [32, 33]. In contrast experiments, the average AUC values and the AUPR values of ten testing sets of each model were gained and the corresponding ROC curves and PR curves were drawn through the results of tenfold cross-validations [34, 35].

### Classifier selection and parameter tuning

After gaining the linear feature matrixes $U$ and $V^{T}$ based on SVD, we found a huge decay gap from $10^{ - 1}$ to $10^{ - 14}$ between the 173rd and the 174th dimensions of the importance matrix $\Sigma$ (Additional file 1). In the light of principle of SVD, the linear features of entities were mainly focused on the top 173 dimensions. Therefore, the linear feature vectors of lncRNA and disease were fixed to 173 dimensions. As node2vec is a highly encapsulated node representation learning method, most of the inner parameters were kept constant and the hyperparameters acted as the dimensions of nonlinear vectors in our model. The16-, 32-, 64-, and 128-dimensional nonlinear feature representations were obtained, respectively.

In the selection process of machine learning classifiers, Linear Regression (LR), Naive Bayes (NB) [36], Random Forest (RF) [37], AdaBoost (ADB) [38] and XGB (XGBoost) [39] were tested based on different integrated features, respectively. The results of ACC and MCC values of all classifiers are shown in Tables 1 and 2. The column named **“SVD”** represents the features extracted based on single SVD method. Analogously, **“N2V16”** represents the 16-dimensional features extracted based on node2vec, **“SN2V16”** represents the integrated features combined with SVD features and 16 dimensional node2vec, and so on. For results on other evaluation indexes Sen, Spec and Prec, refer to Additional file 2, Additional file 3 and Additional file 4 respectively. All above classifiers were imported from scikit-learn library and implemented on Python, all inner-classifier parameters were set as defaults.

**Table 1:** The ACC results of different features on classifiers

	SVD	N2V16	SN2V16	N2V32	SN2V32	N2V64	SN2V64	N2V128	SN2V128
LR	0.9207	0.9381	0.9404	0.9357	0.9370	0.9348	0.9366	0.9389	0.9374
NB	0.8327	0.9189	0.8456	0.9079	0.8559	0.9046	0.8644	0.8978	0.8795
RF	0.9261	0.9288	0.9308	0.9248	0.9303	0.9246	0.9304	0.9257	0.9385
ADB	0.9307	0.9361	0.9376	0.9331	0.9375	0.9289	0.9378	0.9281	0.9350
XGB	0.9383	0.9400	**0.9460**	0.9392	0.9454	0.9365	0.9452	0.9364	0.9444

**Table 2:** The MCC results of different features on classifiers

	SVD	N2V16	SN2V16	N2V32	SN2V32	N2V64	SN2V64	N2V128	SN2V128
LR	0.8412	0.8766	0.8812	0.8717	0.8743	0.8698	0.8736	0.8779	0.8750
NB	0.6719	0.8391	0.6956	0.8717	0.7150	0.8116	0.7317	0.7994	0.7613
RF	0.8527	0.8579	0.8619	0.8500	0.8607	0.8496	0.8609	0.8517	0.8618
ADB	0.8617	0.8705	0.8754	0.8667	0.8752	0.8580	0.8758	0.8566	0.8700
XGB	0.8770	0.8803	**0.8922**	0.8789	0.8920	0.8730	0.8906	0.8730	0.8891

As known from Tables 1 and 2, the combination of linear features and 16-dimensional node2vec features obtained the optimal classification results in the XGBoost classifier (bolded in Tables 1, 2). Moreover, in most classifiers, prediction results based on integrated features were better than single linear feature prediction results and corresponding nonlinear feature prediction results, which demonstrated that the combination of SVD and node2vec does enhance the expression of integrated feature vectors in majority of classifiers.

### Model contrast

**Fig. 1:**
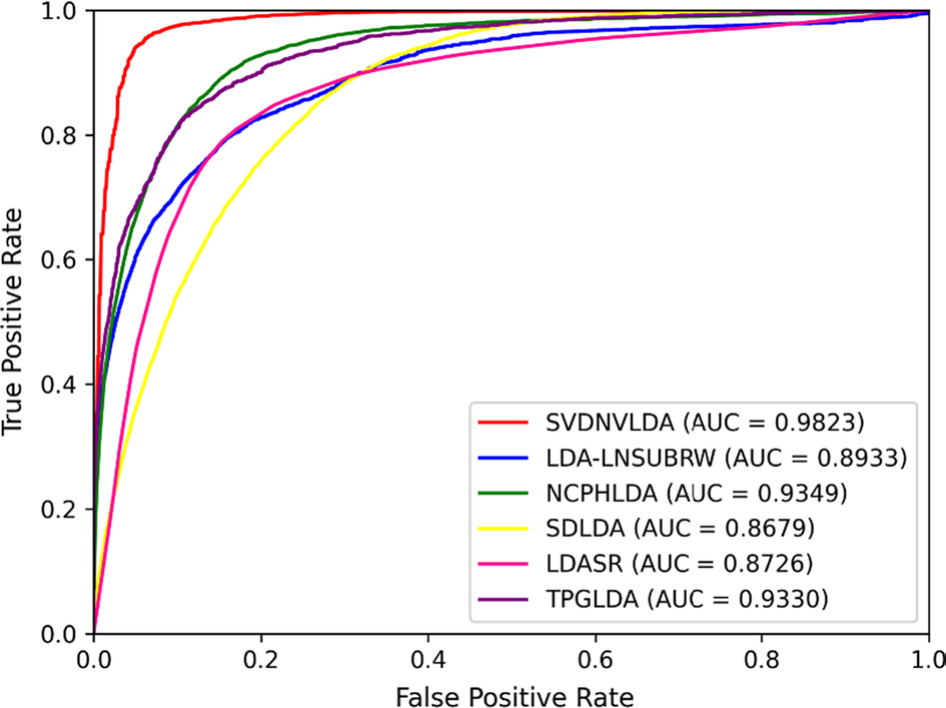
The ROC curves of comparison test

**Fig. 2:**
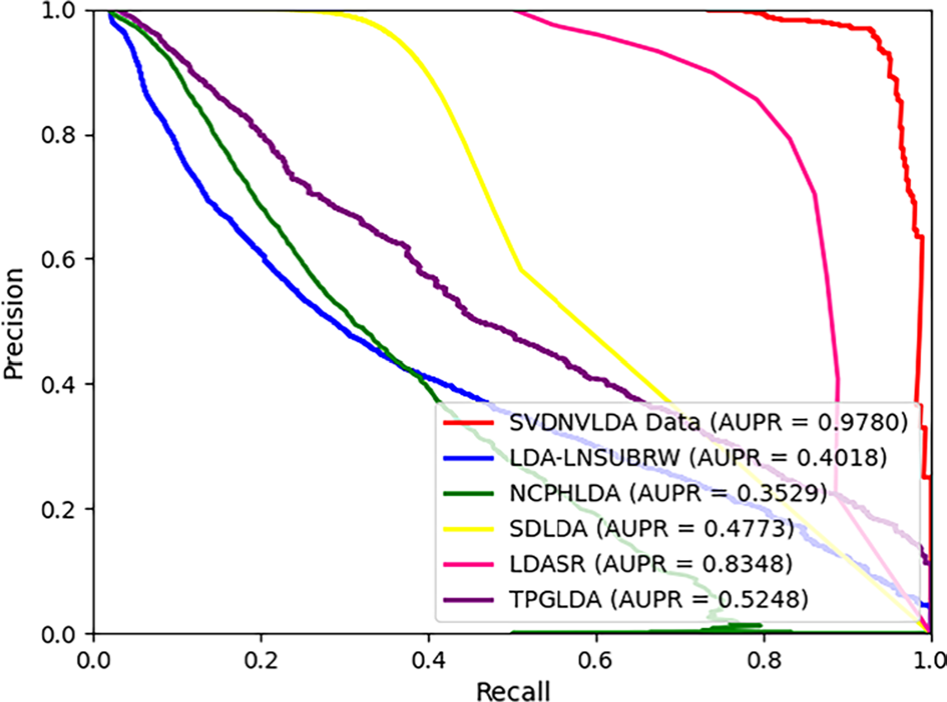
The PR curves of comparison test

After the model construction, we compared the proposed model with five state-of-the-art lncRNA-disease prediction methods: LDASR [25], LDA-LNSUBRRW [22], NCPHLDA [23], SDLDA [28], and TPGLDA [20]. The ROC and PR curves under tenfold cross-validations as well as relevant AUC and AUPR values are shown in Figs. 1 and 2 respectively.

Just as shown in Figs. 1 and 2, both the AUC value and AUPR value of SVDNVLDA are the highest among tested models, which indicated that the outperformance of SVDNVLDA. In terms of AUC, compared with NCPHLDA model, which gained the best result in contrast group, our model also improved the AUC value by about $5{\text{\% }}$. Moreover, the excellent AUPR value manifested that our model also has first-class classification ability on unbalance data sets.

**Fig. 3:**
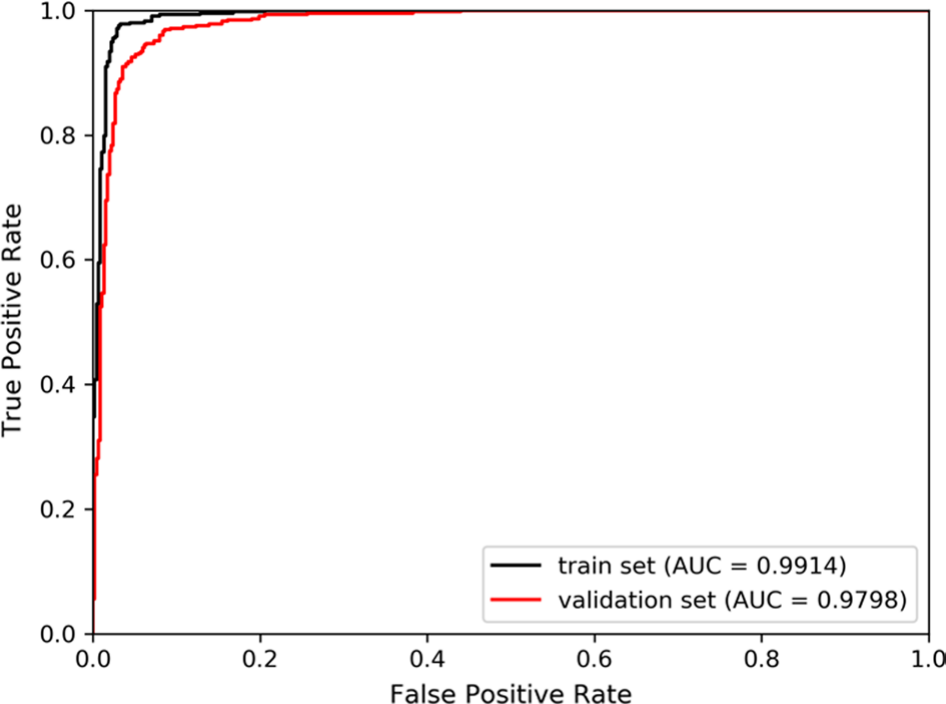
The ROC curves of train set and validation set

**Fig. 4:**
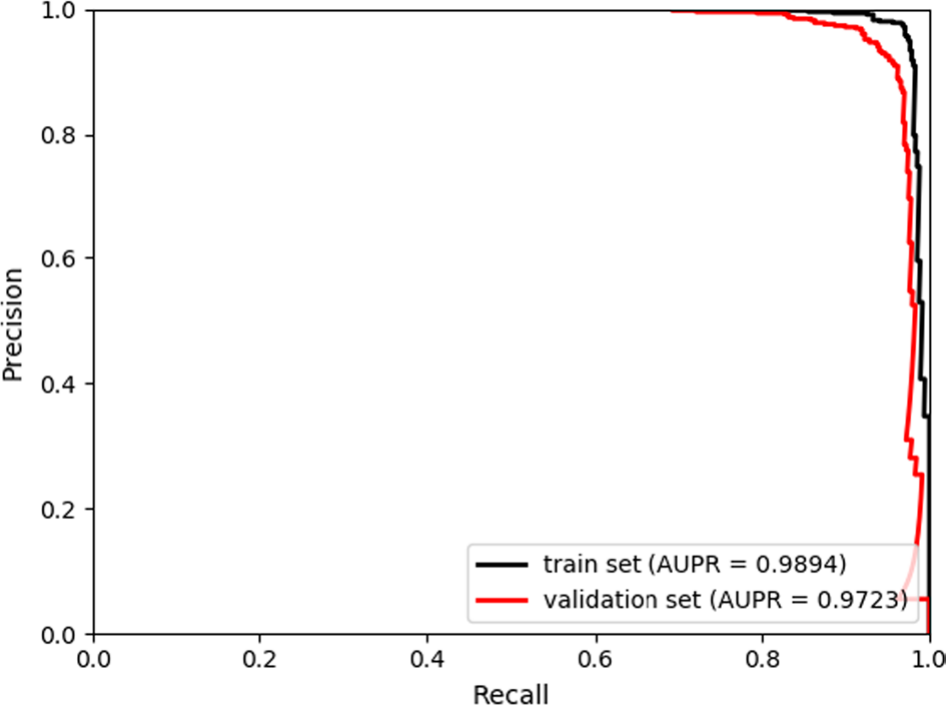
The PR curves of train set and validation set

Since all parameters of XGBoost classifier were set as defaults, to testify whether the AUC and AUPR results of SVDNVLDA is overfitted, we futher seperated 10% samples as validation set and trained classifier without leveraging the validation set. The ROC and PR curves of the train set and the validation set were exhibited in Figs. 3 and 4 respectively. SVDNVLDA achieved remarkable results with AUC of 0.9798 and AUPR of 0.9723 on the validation set, and it was not a result of overfitting.

### Robustness testing

**Fig. 5:**
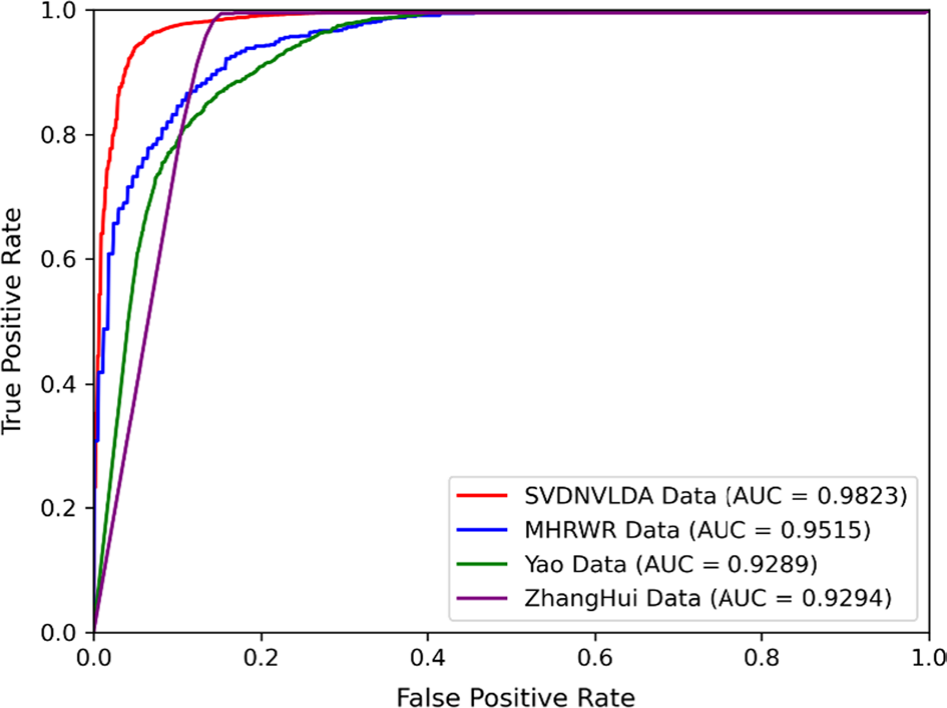
The ROC curves of robustness test

**Fig. 6:**
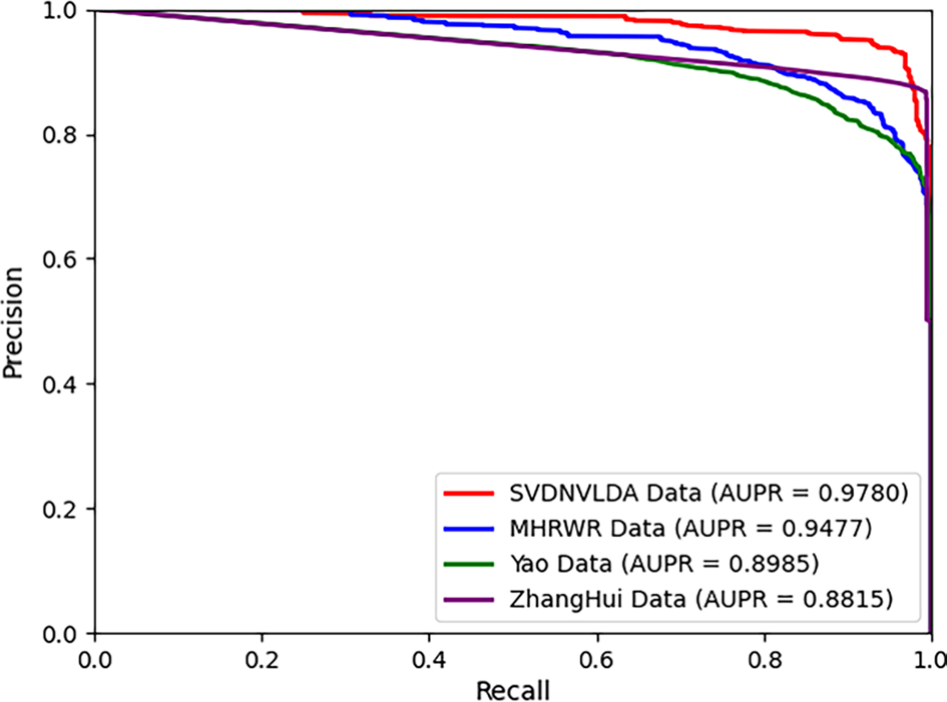
The PR curves of robustness test

The robustness of the predictive model is that the predictive model can give a stable performance for data sets on different scales. For evaluating the robustness of SVDNVLDA, we applied it on three varying scale data sets, which had been adopted by other open-source lncRNA-disease association identification models. Similarly, under tenfold cross validations, the ROC and the PR curves of SVDNVLDA on these data sets are plotted in Figs. 5 and 6 respectively. The data set used in Yao’s model [19] includes 2697 lncRNA-disease associations, 1002 lncRNA-miRNA associations, and 13,562 miRNA-disease associations. And the data leveraged in Zhanghui’s model [40] contains 1151 lncRNA-disease associations, 10,102 lncRNA-miRNA associations and 4634 miRNA-disease associations. While, it is worth mentioning that miRNA entities were replaced with genes in the data set of MHRWR [21], which included 264 lncRNA-gene associations, 855 lncRNA-disease associations, and 9997 gene-disease associations. The experimental test results yielded that SVDNVLDA achieved excellent prediction results on all data sets, in particular, the prediction results after replacing miRNAs with other biological entities were still fine in the MHRWR model data. All these results suggested that SVDNVLDA can be flexible to accommodate data in different scales or even different contents.

### Case studies

To further evaluate the performance of SVDNVLDA model in practical applicaitons, we selected lung cancer, breast cancer and pancreatic cancer as case studies. The general processes of each of case studies were as following: first, all lncRNA-disease association data and the same number of negative samples were utilized to train an XGBoost classifier. Then, all lncRNAs unrelated to the interested disease in experimental data were screened, each of lncRNA feature vectors was combined with the current disease feature vectors. Finally, all these lncRNA-disease feature pairs were inputted into the trained classifier, and the output scores were taken as the correlation probability between the lncRNAs and the corresponding disease. After sorting these scores by descending order, the top ten lncRNA-disease associations were selected. And the validity of selected associations was verified by searching the relative literature in the PubMed database. The results of case studies (Tables 3, 4, 5) and roughly analyses of each disease are as follows.

**Table 3:** Case study results of breast neoplasms

Rank	lncRNA	PMID
1	HCP5	32165090
2	MBNL1-AS1	31113460
3	TNRC6C-AS1	30038597
4	RN7SL1	28709002
5	MIR155HG	32165090
6	DISC1	31783305
7	LRRC2-AS1	*Unknown*
8	NRON	32913541
9	lnc-KCTD6-3	*Unknown*
10	MORT	28690657

**Table 4:** Case study results of lung neoplasms

Rank	lncRNA	PMID
1	TNRC6C-AS1	32041817
2	SNHG16	33015794
3	TUG1	33073961
4	LINC00963	28923857
5	NRON	29772429
6	RN7SL1	*Unknown*
7	lnc-Sox5	23932921
8	HAGLR	28632999
9	LINC00460	32633366
10	AGAP2-AS1	32015683

**Table 5:** Case study results of pancreatic neoplasms

Rank	lncRNA	PMID
1	TNRC6C-AS1	32382761
2	FENDRR	33417179
3	FOXCUT	*Unknown*
4	PCAT1	33629282
5	MBNL1-AS1	*Unknown*
6	lnc-KCTD6-3	32046932
7	MIR31HG	32134327
8	CASC9	33520364
9	DGCR5	33613108
10	MORT	26549028

[Breast Cancer] According to the latest data of the global cancer burden in 2020 [41], there were 2.26 million new cases of Breast cancer worldwide in 2020, accounting for 11.7% of all new cases of cancer this year, ranking first among all cancers. Symptoms of breast cancer includes lumps in the breast, changes in the shape of the breast, depressions in the skin with bone pain, swollen lymph nodes, tachypnea or yellow skin. Table 3 shows the top-10 lncRNA-disease associations of unknown association of SVDNVLDA for breast cancer prediction.

[Lung Cancer] Lung cancer is a kind of malignant lung tumor caused by uncontrolled cell growth in lung tissues, the malignant growth can spread beyond the lungs by metastasizing to nearby tissues or other parts of the body. In 2020, there were 2.2 million new cases of lung cancer worldwide, accounting for 11.4% of all the new cancer cases, ranking secondly among all cancers [41]. The most common symptoms of lung cancer include coughing, weight loss, breath hard and chest pain. Most of lung cancer cases are caused by long-term smoking. Table 4 illustrates the top-10 lncRNA results of lung cancer predicted by SVDNVLDA.

[Pancreatic Cancer] The common signs and symptoms of pancreatic cancer include yellow skin, abdominal or back pain, unexplained weight loss and loss of appetite. Usually, there are no obvious symptoms in the early stages of pancreatic cancer, yet when the symptoms are sufficient to indicate contraction generally means the disease is at an advanced stage, and by the time of diagnosis, pancreatic cancer has usually spread to other parts of the body. In the global statistics of cancer deaths in 2020, pancreatic cancer caused 466,000 deaths, and more than half of these clinical cases of pancreatic cancer were over 79 years old [41]. Table 5 presents the top-10 potential lncRNAs with pancreatic cancer prediction.

Among all the results of three diseases, the latest Pubmed literature support was found for 8, 9 and 8 of the top-10 predicted lncRNAs with maximum correlation probability, respectively. This clearly indicates that our model has a good performance in the prediction of actual disease-related lncRNAs, and possess potential application value and scientific significance. Full results of the three cancers are given in Additional file 5, Additional file 6 and Additional file 7.

## Discussions

In this paper, we proposed an integrated feature extraction model, SVDNVLDA, for predicting potential lncRNA-disease associations. In SVDNVLDA, the network representation learning method node2vec and matrix decomposition method SVD were originally integrated to predict the potential lncRNA-disease associations. It also can be regarded as an open framework, in which more feature extraction methods can be flexibly applied.

However, there are still some potential weaknesses in our model, which mainly relies on the limitations of the data used in this paper. Specifically, relying solely on the associated data almost could not comprehensively reflect the complex interactions between lncRNAs and the other biomolecules. Meanwhile, in the heterogeneous network LMDN, the node representations, obtained by node2vec, have been proven to be capable to retain the topology information of nodes in network, yet they fail to remain the information of different node types which is abundant and valuable in heterogeneous networks. It would be improved on the expansion of experimental data and introducing more advanced representation learning methods in future studies.

## Conclusions

In SVDNVLDA, the linear feature representations of lncRNAs and diseases containing their linear interaction information were obtained by matrix decomposition method SVD; and the nonlinear features containing network topology information were obtained by node2vec. The integrated feature vectors of aforementioned features were inputted into a machine learning classifier, which transformed the lncRNA-disease association prediction into a binary classification problem. The AUC and AUPR values of SVDNVLDA are higher than any of five popular prediction methods under tenfold cross-validations. The prediction performance on data sets of different scales shows that SVDNVLDA can be adapted to a range of data sets and possess strong robustness. In addition, the case studies of three common cancers indicate its effectiveness in practical applications.

## Materials and methods

### Overview of SVDNVLDA

**Fig. 7:**
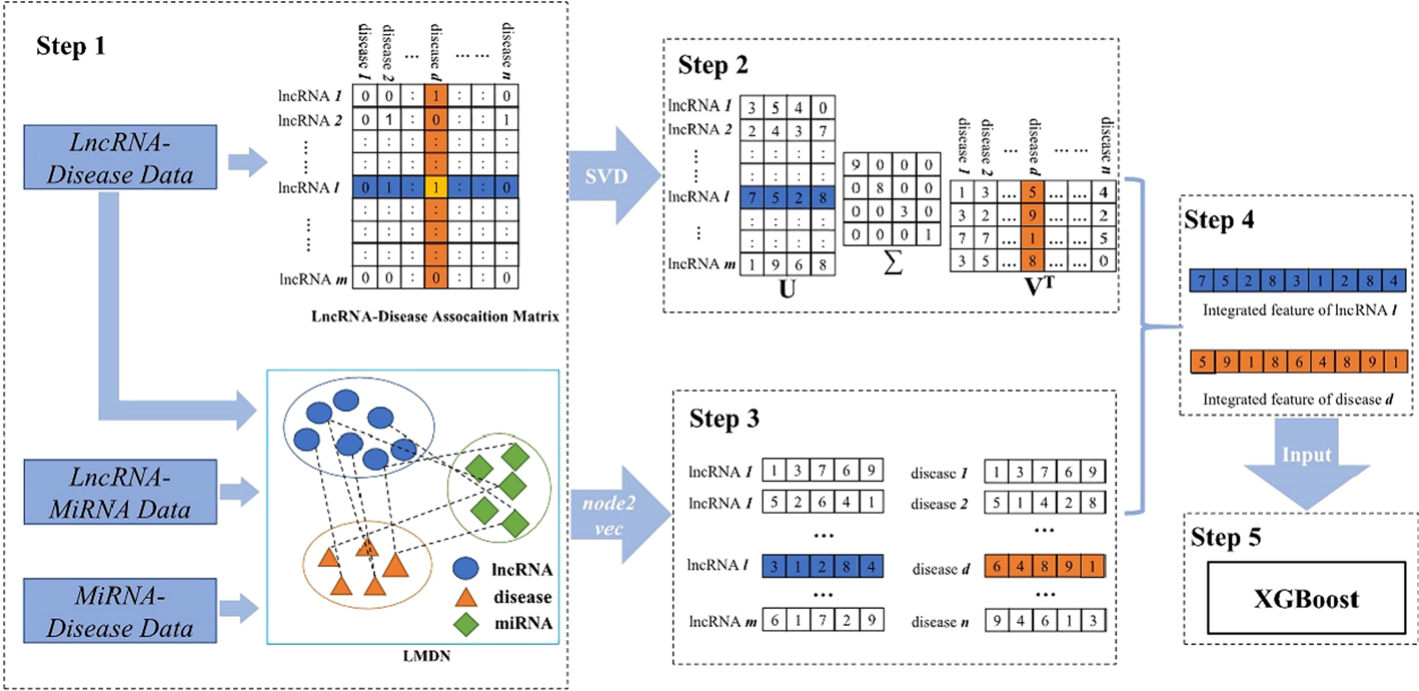
The flowchart of SVDNVLDA

Matrix decomposition method, SVD, and network embedding method, node2vec, were novelly integrated in SVDNVLDA for obtaining the linear and the nonlinear representations of both lncRNA and disease entities respectively. By combining the different features of each lncRNA and each disease, the integrated feature vectors were constructed which fused the linear features of interaction information and the nonlinear features of network topology information. These feature vectors were served as the inputs of one machine learning classifier and the corresponding predicted results would be obtained in the end (Fig. 7).

Step1: Data processing and construction of lncRNA-disease association matrix and lncRNA-miRNA-disease association network (LMDN). Step 2: Apply SVD on association matrix to get linear features. Step 3: Apply *node2vec* on LMDN to get nonlinear features. Step 4: Feature integration. Step 5: Use XGBoost classifier to predict association.

### Data preprocessing

The study mainly included lncRNA-disease association data, lncRNA-miRNA association data and miRNA-disease association data. The experimentally confirmed lncRNA-disease association data were downloaded from LncRNADisease v2.0 [42] and Lnc2Cancer v3.0 [43]. All disease names were converted into standard MESH disease terms, and duplicate data was filtered to retain only one replication. For avoiding experimental errors that came from the downloaded data, the lncRNAs with one or none association were removed. In the end, a total number of 4518 associations between 861 lncRNAs and 253 diseases were obtained.

The known lncRNA-miRNA association data was downloaded from Encori [44] and NPInter V4.0 databases [45]. After eliminating redundancy, only records of the lncRNAs commonly to lncRNA-disease data and the miRNAs commonly to miRNA-disease data were selected. Finally, a total of 8172 lncRNA-miRNA associations were obtained involving 338 lncRNAs and 285 miRNAs.

As for miRNA-disease association data, it was obtained from the HMDD v3.2 database [46]. The original data includes two types of association records, namely the subjective causality and passive changes of miRNAs during the course of diseases. By contrast, the studies of miRNAs in causal relationship with diseases were more valuable for exploring the pathogenesis and searching for new biomarkers. In our experiment, only the related records with causal relationships in HMDD database were picked. All disease names were transformed to standardized names based on MeSH glossary, and the lncRNAs associated with only one disease were removed from the original data. Ultimately, a total count of 861 lncRNAs, 437 miRNAs and 431 diseases were involved in our experiment. The statistical overview of formed data, also as the statistical overview of LMDN was documented in Additional file 8.

### Construct lncRNA-disease association matrix and LMDN

Firstly, the lncRNA- disease association matrix was constructed. For lncRNA $l$, if there is a known association with disease $j$ in our collected data, the corresponding element value in the association matrix $R_{M \times N}$ is $1$; otherwise, it is $0$. The formula is made out as:

1 $${\text{R}}_{{{\text{M}} \times {\text{N}}}} \left( {\text{i,j}} \right){ = }\left\{ {\begin{array}{*{20}l} {1,} \hfill & {{\text{if}}\,{\text{i}}\,{\text{and}}\,{\text{j}}\,{\text{have}}\,{\text{association}}} \hfill \\ {0,} \hfill & {{\text{otherwise}}} \hfill \\ \end{array} } \right.$$

in our experiment, the real matrix ${\text{R}}_{{{\text{M}} \times {\text{N}}}}$ was shaped as 861 × 437 dimensions.

After the construction of association matrix, lncRNA-disease association data combined with lncRNA-miRNA association and miRNA-disease association data were used to construct lncRNA-miRNA-disease association heterogeneous network (LMDN). Among the three types of vertices in LMDN, namely lncRNA, miRNA and disease, there would be an edge between two vertices with association record, otherwise the two vertices would have no connection. The heterogeneous network was a sparse network with 1769 nodes and 16,878 edges, as detailed in Additional file 8.

### Linear feature extraction based on singular value decomposition

SVD is a matrix decomposition method which has been widely used in recommender systems [47, 48]. In SVD, the matrix is common decomposed into the multiplying of three matrices:

2 $$R_{M \times N} = U_{M \times C} \cdot \Sigma_{C \times C} \cdot V_{C \times N}^{T}$$

As a typical collaborative filtering-based recommendation system with SVD, the initial matrix $R$ represents a rating matrix for $M$ users’ rates on $N$ goods. Among the resulted matrixes, $U$ represents the interesting levels of $M$ users on $C$ features of goods, namely users’ characteristics or commodity affinity; while $\Sigma$ represents the importance of each feature of goods, specified as a non-negative diagonal matrix, in which diagonal elements are arranged as descending order. $V^{T}$ represents the distribution of $C$ features in $N$ goods [49].

Analogically, applying SVD on lncRNA-disease association matrix $R_{M \times N}$, the obtained matrixes $U$, $\Sigma$ and $V^{T}$ could represent lncRNA feature matrix, feature weight matrix and disease feature matrix, respectively. For dimensional reduction purpose, only the ranked $k$ features with larger numerical values in $\Sigma$ were taken, and $R$ would be expressed as:

3 $$R_{M \times N} \approx U_{M \times k} \cdot {\Sigma }_{k \times k} \cdot V_{k \times N}^{T}$$

**Fig. 8:**
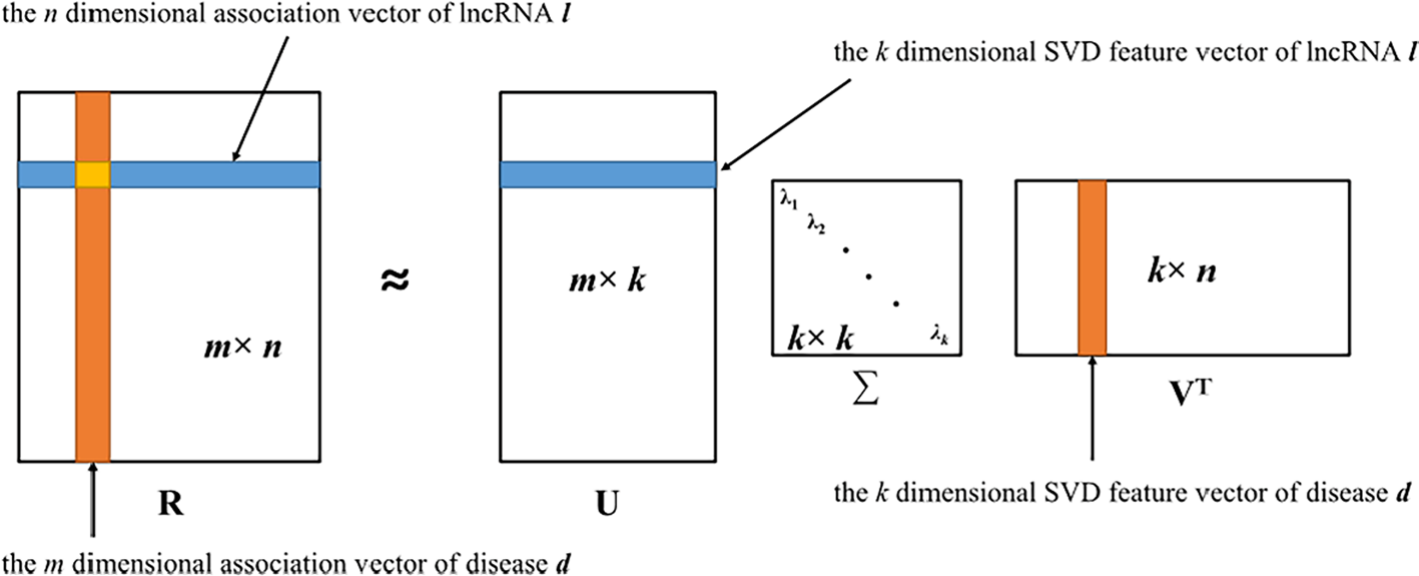
The illustration of applying SVD on lncRNA-disease association matrix

In fact, the binary matrix $R$ is not an ideal initial matrix. In recommendation system, $0$ (or blank) elements in rating matrixs cannot actually represent these rates of products, more likely, it is commonly due to missing users’ evaluations. Thus, in lncRNA-disease association matrix $R$, the value $0$ usually represents that the corresponding association has not been confirmed. Therefore, for calculation convenience and considering biological meaning, all the $0$ elements in original binary matrix $R$ were replaced by $10^{ - 6}$ in our experiment. Based on the theory of SVD, each row in $U_{M \times k}$ represents a $k$-dimensional linear feature vector of a certain lncRNA. Similarly, each column in $V_{k \times N}^{T}$ represents a $k$-dimensional linear feature vector of a certain disease (Fig. 8).

### Nonlinear feature extraction based on *Node2vec*

Network representation learning (NRL), also known as network embedding, refers to map nodes into a continuous low-dimensional space on the premise of keeping characteristics of nodes in the original network. Given a network $G = \left( {V,E} \right)$, where $V = \left\{ {v_{i} } \right\}$ represents the collection of nodes and $E = e_{i} \subset \left\{ {V \times V} \right\}$ represents the collection of edges. The mathematical expression of NRL is: $\forall v_{i}$, find a map $f:V \to R^{d}$, and $d \ll \left| V \right|$. The ideal learned node representations should be able to quantify the characteristics of nodes in social network, which could be intuitively expressed that topological neighbor nodes have small numerical vector distance and the representations of nodes in the same community have larger similarity than nodes outside the community. Up to now, many NRL methods have been widely used to solve problems such as node classification, community discovery, link prediction and data visualization [50].

**Fig. 9:**
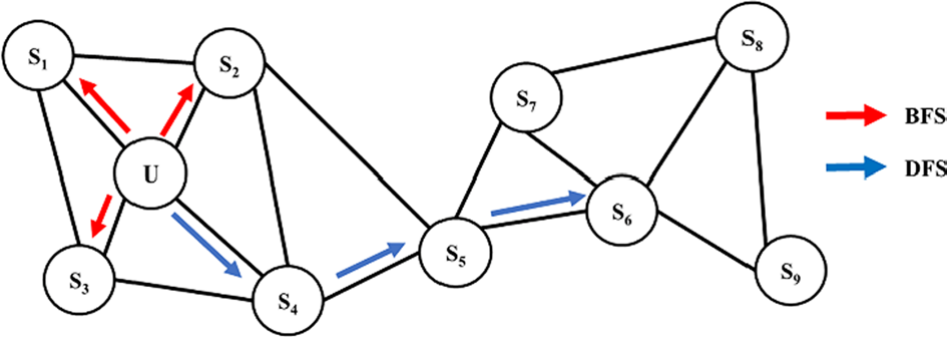
The illustration of distinctions between BFS and DFS

**Fig. 10:**
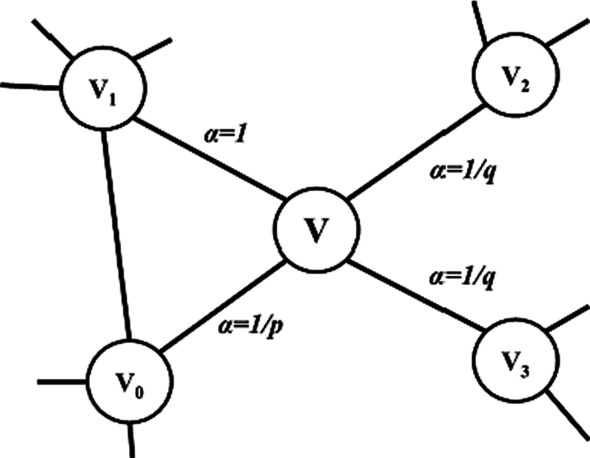
The bias random walk on *node2vec*

As a semi-supervised network feature learning method, node2vec [51] innovatively proposed a biased random walk on the basis of word representation method [52] and DeepWalk [53], as well as defined a more flexible way to select the next step node with random walk. More specifically, node2vec trades off the two kinds of random walk strategy: Breadth-first search (BFS) and Depth-first search (DFS), which are shown in Fig. 9. Unlike the original random walk, node2vec can artificially control the degree of BFS and DFS by adjusting parameters based on the preferences of actual practice scenario. Here is a detailed description of simple random walk and modified biased random walk in node2vec (Fig. 10).

For a given boot node $u$, simulate a simple unbiased random walk with $l$ length. $c_{i}$ represents the $i^{th}$ node in the process of random walk. Let $c_{0} = u$, and the transition probability of the node reached in $i^{th}$ step is:

4 $${\text{P(c}}_{{\text{i}}} = {\text{x|c}}_{{{\text{i}} - {1}}} = {\text{v)}} = \left\{ {\begin{array}{*{20}l} {\frac{{\uppi _{{{\text{vx}}}} }}{{\text{Z}}}{,}} \hfill & {{\text{if}}\,{\text{(v,x)}} \in {\text{E}}} \hfill \\ {0,} \hfill & {{\text{otherwise}}} \hfill \\ \end{array} } \right.$$

of which $\pi_{vx}$ is the unnormalized transition probability between nodes $v$ and $x$, $Z$ represents a normalized constant term.

As for the biased random walk in node2vec, just as shown in Fig. 10, if the root position of a random walk is set at node $t$, through edge $\left( {t,v} \right)$, the current position reached node $v$, and the transition probability is set as follows:

5 $$\alpha_{pq} \left( {t,x} \right) = \left\{ {\begin{array}{*{20}l} \frac{1}{p} \hfill & { if\,d_{tx} = 0} \hfill \\ 1 \hfill & {if\,d_{tx} = 1} \hfill \\ \frac{1}{q} \hfill & { if\,d_{tx} = 2} \hfill \\ \end{array} } \right.$$

$d_{tx}$ represents the shortest distance between nodes $t$ and $x$ and the possible value of $d_{tx}$ is 0,1,2. As shown in Fig. 10, the parameter $p$ controls the probability that the next step of walk will return to the previous node. If $p$ is greater than $1$, the random walk will have less tendency to turn back. The value of $q$ controls the preference of BFS and DFS to guide the bias of random walk. If $q$ is greater than $1$, the random walk will be more inclined to BFS, that is, to the neighbor node of the starting node. If $q$ is less than $1$, the random walk is more inclined to DFS, that is, to go away from the starting node. When the values of $p$ and $q$ are both equal to $1$, node2vec is equal to DeepWalk.

In the constructed LMDN, node2vec was adopted to obtain the corresponding representations for vertices. The representations of lncRNA and disease nodes generated by node2vec retain the topological information of the nodes in LMDN. The experimental results demonstrate that the obtained nonlinear features could effectively enhance the SVD based linear features and improve the information richness in integrated features.

### Feature integration

Based on the decomposition of $R_{M \times N}$ and NRL method node2vec, we have obtained the linear feature matrixes $U$, $V^{T}$, and the nonlinear feature representations of lncRNA and disease nodes in LMDN. For each lncRNA $i$ and disease $j$, the feature integration rules are as follows:

The linear features corresponding to lncRNA $i$ is the *i*th row of $U$, which is noted as $LL_{i}$ after being converted into a column vector. Similarly, the linear features corresponding to disease $j$ is the *j*th column of $V^{T}$, represented as $LD_{j}$. The nonlinear features corresponding to $i$ is noted as $NL_{i}$ as well as the nonlinear features corresponding to $j$ is noted as $ND_{j}$. The final integrated features of $i$ and $j$ is expressed as:

6 $$FL_{i} = \left[ {\begin{array}{*{20}c} {LL_{i} } \\ {NL_{i} } \\ \end{array} } \right]$$

7 $$FD_{j} = \left[ {\begin{array}{*{20}c} {LD_{j} } \\ {ND_{j} } \\ \end{array} } \right]$$

where [] represents the vector connect operation.

## Supplementary Information

**Additional file 1**. The numerical values of diagonal elements in the importance matrix Σ.

**Additional file 2**. The sensitivity results of different features in classifiers.

**Additional file 3**. The specificity results of different features in classifiers.

**Additional file 4**. The precision results of different features in classifiers.

**Additional file 5**. The complete case study results of breast cancer.

**Additional file 6**. The complete case study results of lung cancer.

**Additional file 7**. The complete case study results of pancreatic cancer.

**Additional file 8**. The statistical overview of experimental data.

## Data Availability

The source code and data of SVDNVLDA are available at https://github.com/iALKing/SVDNVLDA.

## Abbreviations

SVD: Singular Value Decomposition
ncRNAs: Non-coding RNAs
RWR: Random walk with restart
LRLSLDAL: Laplacian Regularized Least Squares for lncRNA-Disease Association
SVDNVLDA: Singular Value Decomposition and Node2Vec based LncRNA-Disease Association prediction model
Acc: Accuracy
Sen: Sensitivity
Spec: Specificity
Prec: Precision
MCC: Matthews correlation coefficient
LR: Linear regression
NB: Naïve Bayes
RF: Random Forest
ADB: AdaBoost
XGB: XGBoost
LMDN: LncRNA-miRNA-disease interaction heterogeneous network
NRL: Network representation learning
BFS: Breadth-first search
DFS: Depth-first search
